# Estimating risk of mechanical ventilation and in-hospital mortality among adult COVID-19 patients admitted to Mass General Brigham: The VICE and DICE scores

**DOI:** 10.1016/j.eclinm.2021.100765

**Published:** 2021-02-25

**Authors:** Christopher J. Nicholson, Luke Wooster, Haakon H. Sigurslid, Rebecca H. Li, Wanlin Jiang, Wenjie Tian, Christian L. Lino Cardenas, Rajeev Malhotra

**Affiliations:** a Cardiovascular Research Center, Division of Cardiology, Department of Medicine, Massachusetts General Hospital, Yawkey 5700; 55 Fruit Street, Boston, MA 02114, United States; b Case Western Reserve University School of Medicine, Cleveland, Ohio, United States; c Department of Cardiology, Sichuan Academy of Medical Sciences & Sichuan Provincial People's Hospital, School of Medicine, University of Electronic Science and Technology of China, Chengdu, Sichuan Province, China

## Abstract

**Background:**

Risk stratification of COVID-19 patients upon hospital admission is key for their successful treatment and efficient utilization of hospital resources. We sought to evaluate the risk factors on admission (including comorbidities, vital signs, and initial laboratory assessment) associated with ventilation need and in-hospital mortality in COVID-19.

**Methods:**

We established a retrospective cohort of COVID-19 patients from Mass General Brigham hospitals. Demographic, clinical, and admission laboratory data were obtained from electronic medical records of patients admitted to the hospital with laboratory-confirmed COVID-19 before May 19, 2020. Multivariable logistic regression analyses were used to construct and validate the Ventilation in COVID Estimator (VICE) and Death in COVID Estimator (DICE) risk scores.

**Findings:**

The entire cohort included 1042 patients (median age, 64 years; 56.8% male). The derivation and validation cohorts for the risk scores included 578 and 464 patients, respectively. We found four factors to be independently predictive for mechanical ventilation requirement (diabetes mellitus, SpO_2_:FiO_2_ ratio, C-reactive protein, and lactate dehydrogenase), and 10 factors to be predictors of in-hospital mortality (age, male sex, coronary artery disease, diabetes mellitus, chronic statin use, SpO_2_:FiO_2_ ratio, body mass index, neutrophil to lymphocyte ratio, platelet count, and procalcitonin). Using these factors, we constructed the VICE and DICE risk scores, which performed with C-statistics of 0.84 and 0.91, respectively. Importantly, the chronic use of a statin was associated with protection against death due to COVID-19. The VICE and DICE score calculators have been placed on an interactive website freely available to healthcare providers and researchers (https://covid-calculator.com/).

**Interpretation:**

The risk scores developed in this study may help clinicians more appropriately determine which COVID-19 patients will need to be managed with greater intensity.

**Funding:**

COVID-19 Fast Grant (fastgrants.org).

**Research in Context**

**Evidence before this study**

The precise clinical risk factors associated with poor in-hospital outcomes for patients with COVID-19 remain incompletely elucidated. Early studies on COVID-19 highlighted the presence of cardiovascular comorbidities and increased inflammatory markers as predictors of poor outcomes. However, a comprehensive assessment of clinical, laboratory, and hemodynamic risk factors at the time of hospital admission to aid in the risk stratification of patients with COVID-19 was needed. An algorithm developed from a study of Chinese patients was developed that predicted critical illness (a composite of ICU admission, ventilation needs and death) in hospitalized COVID-19 patients, however distinct risk scores for the prediction of mechanical ventilation requirement or for in-hospital mortality were not available.

**Added value of this study**

In our study, we assessed the baseline comorbidities, presenting clinical, laboratory, and hemodynamic findings, and outcomes of hospitalized COVID-19 patients admitted to Mass General Brigham hospitals in Boston, Massachusetts and developed multivariable risk models to more effectively predict severe outcomes in COVID-19 patients. This original research article used a derivation and independent validation cohort to construct a risk score for need for mechanical Ventilation In COVID-19 Estimator (VICE score) and a risk score for in-hospital Death In COVID-19 Estimator (DICE score) in over 1000 patients hospitalized for COVID-19.  Because these risk scores incorporate factors unique to COVID-19, they were found to provide enhanced accuracy for risk prediction compared to a modified SOFA score.

**Implications of all the available evidence**

It is crucial for health care providers to be able to stratify risk for the most important clinical outcomes in COVID-19, namely mechanical ventilation need and mortality. The available evidence indicates that several unique risk factors exist for critical illness and mortality in COVID-19. Precise quantification of the risk of these outcomes will allow the most optimal allocation of health care resources on admission to the hospital and identify those that will require the most intense care.

Alt-text: Unlabelled box

## 1 Introduction

The number of global confirmed cases of the novel coronavirus disease 2019 (COVID-19) passed 75 million in December 2020, with over 1.6 million deaths [1]. The U.S. has surpassed any other country in the number of total deaths and case rates continue to rise with some hospitals utilizing nearly 100% of available intensive care unit (ICU) beds.

Specific information regarding the patient risk factors that associate with mortality from COVID-19 remain limited, and methods to accurately predict severity of disease at the time of hospital presentation are lacking [[2], [3], [4], [5], [6]]. Using data from a Chinese cohort, an algorithm was developed that predicts critical illness (a composite of ICU admission, ventilation needs and death) in hospitalized COVID-19 patients. [7]. However, the applicability of this algorithm to predict outcomes in a US population, which has distinct disease risk profiles, remains unknown [7,8]. Further, it is crucial for health care providers to be able to stratify risk for the most important clinical outcomes in COVID, namely mechanical ventilation need and mortality. Knowing the risk of these outcomes will allow the most optimal allocation of health care resources on admission to the hospital and identify those that will require the most intense care. Given the United States has reported approximately one fifth of global deaths due to COVID-19 and is currently in the midst of a profound wave of infections, new information on the factors that influence risk of severe outcomes is greatly needed.

This study investigates patients with laboratory confirmed COVID-19 admitted to Mass General Brigham hospitals in Boston, Massachusetts. Specifically, we described the baseline comorbidities, presenting clinical tests and outcomes of hospitalized COVID-19 patients, explored the risk factors on admission associated with mechanical ventilation requirements and in-hospital death, and developed risk models to more effectively predict severe outcomes in patients from the United States.

## 2 Methods

### 2.1 Study population

This study included consecutive adult patients with laboratory-confirmed COVID-19 infection who were admitted for illness related to COVID-19 to five hospitals in the Mass General Brigham health care system (Massachusetts General Hospital, MGH; Brigham and Women's Hospital, BWH; Newton Wellesley Hospital, NWH; Brigham and Women's Faulkner Hospital, BWFH; and North Shore Medical Center, NSMC) in the Boston region before May 19, 2020. This date was chosen to allow a large enough sample size (*n*>1000) while still providing complete hospital outcome data. Decisions to admit to hospital were made subjectively by the assessing clinicians at point of referral. The Mass General Brigham institutional review board (IRB) approved the study (Protocol # 2020P000982). The need for informed consent was waived by the IRB as all patient data was obtained from the electronic medical records and the study did not present any risk to subjects.

A confirmed case of COVID-19 was defined by a positive result on a reverse-transcriptase–polymerase-chain-reaction (RT-PCR) assay of a specimen collected on a nasopharyngeal swab. Patients were defined as COVID-19 positive if they had a positive test, or if they had a negative test, but repeat testing was positive. Only laboratory-confirmed cases of those that were sufficiently ill to require hospital admission were included. Clinical outcomes were monitored up to hospital discharge. We excluded children (those younger than 18 years of age) from the study.

### 2.2 Data collection

Epidemiological, demographic (self-reported), clinical, laboratory, and home medication data were obtained from the Research Patient Data Repository (RPDR), a centralized clinical data registry directed by the Mass General Brigham network [9]. Outcome data, including discharge, ICU, and ventilation status were extracted from electronic medical records (EPIC) using a standardized data collection form [10]. All laboratory tests and radiologic assessments, including plain chest radiography, were performed at the discretion of the treating physician. Only laboratory tests performed on or within 24 h of hospital admission were included in the analyses. Patients were assessed for the presence of hypertension, diabetes mellitus, coronary artery disease (CAD), chronic obstructive pulmonary disease (COPD), chronic kidney disease (CKD), and a history of cancer. These covariates were selected based on their significant association with clinical outcomes in COVID-19 patients, as described in previously published analyses.[2,3,[5], [6], [7],^11^].

### 2.3 Outcome definitions and data analysis

The primary endpoints for our analyses were need for mechanical ventilation and in-hospital death. For our statistical analyses, we excluded patients that requested, upon admission, to be treated with comfort measures only (CMO). Patients that were identified as “do not intubate” (DNI) on admission were excluded from analyses that assessed risk factors for mechanical ventilation requirements. Discharged patients were defined as those who were discharged to home, nursing homes, or a rehabilitation facility. Continuous variables were reported as mean (SEM) unless otherwise noted and categorical variables were reported as n (%). The Fisher's exact test, Student's *t*-test or Mann Whitney test were used to measure differences between groups, where appropriate. Univariate logistic regression was used to determine if a clinical factor was associated with the need for mechanical ventilation or with in-hospital mortality.

### 2.4 Multivariable logistic regression and risk score construction

We used a multivariable logistic regression model to determine variables that would be included in our predictive risk score algorithms for mechanical ventilation needs (Ventilation in COVID Estimator [VICE] score) and in-hospital death (Death in COVID Estimator [DICE] score). To do so, we determined predictive variables in a derivation cohort and then validated these findings in an independent validation cohort. Since MGH admitted the most patients, we used this as our derivation cohort, and patients admitted to BWH, NWH, BWFH, or NSMC served as our validation cohort. For variables that had a univariate P-value of <0.05 or a P-value <0.05 after adjusting for age and sex in this cohort, we performed a multivariable logistic regression analysis with a backwards stepwise approach, retaining all variables with a *p*<0.05. Of variables that were highly correlative (e.g. estimated glomerular filtration rate [eGFR] and creatinine, with *r* >0.7), we only included the one with the lowest p-value in univariate analysis. Laboratory markers that exhibited a skewed distribution were log_2_-transformed for the regression analysis. We used this method to determine risk factors independently associated with (i) mechanical ventilation requirements and (ii) in-hospital mortality, which allowed construction of risk score predictors for each outcome. We assessed the accuracy of the risk score models using the area under the receiver-operator characteristic curve (AUC or C-statistic). We first assessed our risk score in patients that were admitted to MGH only (our derivation cohort), and using the same beta coefficients as weighting factors, then validated it using patients admitted to BWH, NWH, BWFH, and NSMC (our validation cohort). Differences in in-hospital outcomes across racial groups were studied as well as differences in the DICE and VICE risk scores. Because patients who were CMO on admission were excluded from analysis (and those who were DNI were excluded from analyses of mechanical ventilation), we assessed whether differences in the proportion of CMO or DNI status across racial groups were present. Statistical analyses were performed using Stata (version 13.0) and graphs were constructed with R studio (version 1.2.5033).

This study adheres to the Strengthening the Reporting of Observational Studies in Epidemiology (STROBE) guidelines (https://www.equator-network.org/reporting-guidelines/strobe/).

### 2.5 Role of funding sources

The funders of the study had no role in study design, data collection, data analysis, data interpretation, or writing of the report. The corresponding authors had full access to all the data in the study and had final responsibility for the decision to submit for publication.

## 3 Results

### 3.1 Demographic and clinical characteristics of the cohort

1137 adults were admitted to Mass General Brigham hospitals with COVID-19 symptoms before May 19, 2020. Patients that were treated with comfort measures only (CMO) on arrival (*n* = 95) to the hospital were excluded from the study. As described in **Supplemental Table 1**, CMO patients were on average older and had a higher level of cancer diagnoses than patients included in the final study. After this exclusion, we included 1042 patients (578 from MGH, 269 from BWH, 125 from BWFH, 60 from NWH, and 10 from NSMC) in our final analyses (Table 1). The median age for these patients was 64 (IQR: 53–75, Range = 18–99), and the majority (56.8%) were male. Among the 1042 patients, 438 (42%) identified as white, 187 (17.9%) as Black, 113 (10.8%) as Hispanic, and 37 (3.6%) as Asian. One hundred and seventy-seven (17%) patients either identified with a mix of racial backgrounds or did not identify with any of these racial backgrounds (Other/Mix) and 90 (8.6%) patients had no racial background recorded. Three quarters of the patients had at least one comorbidity. The most common comorbidities were hypertension (56.4%), and diabetes mellitus (42.5%, Table 1). With regards to long-term home medications (prior to COVID-19 admission), 511 (49.0%) were on a statin, 318 (30.5%) on aspirin, 315 (30.2%) on a renin-angiotensin-aldosterone system (RAAS) inhibitor, and 44 (4.2%) were on an anticoagulant.

**Table 1:** Baseline characteristics of hospitalized Covid-19 patients included in the study.

Characteristic	All patients	Ventilation Status				Mortality			
		Not ventilated	Ventilated	OR (95% CI)	P-Value	Discharged	Deceased	OR (95% CI)	P-Value
Total patients	1042	547	404		–	832	210		–
Median (IQ) Age (years)	64 (53–75)	61 (51–72)	64 (53–72)	1.07 (0.98–1.16)	0.12	61 (50–71)	76 (66–82)	2.03 (1.78–2.32)	<0.001
Male Sex, No. (%)	592 (56.8)	284 (51.9)	264 (65.3)	1.75 (1.34–2.28)	<0.001	453 (54.4)	139 (66.2)	1.64 (1.20–2.26)	0.002
**Admission vital signs, Mean (SEM)**									
Weight (kg)	83.9 (0.7)	83.5 (0.9)	86.0 (1.0)	1.01 (1.00–1.01)	0.075	84.4 (0.7)	82.1 (1.5)	0.99 (0.99–1.00)	0.162
BMI (kg/m^2^)	29.9 (0.2)	29.8 (0.3)	30.4 (0.3)	1.01 (0.99–1.03)	0.2	30.1 (0.2)	29.1 (0.5)	0.98 (0.95–1.00)	0.052
Temperature > 38°C, No. (%)	159 (15.3)	71 (13.0)	82 (20.3)	1.16 (1.00–1.36)	0.057	129 (15.5)	30 (14.3)	0.89 (0.74–1.08)	0.247
HR (beats/min)	87.3 (0.6)	86.1 (0.7)	89.1 (1.0)	1.01 (1.00–1.02)	0.013	86.9 (0.6)	88.8 (1.5)	1.01 (1.00–1.01)	0.181
Systolic BP, mmHg	128.7 (0.7)	130.8 (0.9)	126.5 (1.2)	0.99 (0.99–1.00)	0.004	129.3 (0.8)	126.0 (1.6)	0.99 (0.99–1.00)	0.059
Diastolic BP, mmHg	70.1 (0.4)	71.8 (0.5)	68.6 (0.6)	0.98 (0.97–0.99)	<0.001	70.8 (0.4)	67.7 (0.9)	0.98 (0.96–0.99)	0.001
Respiratory Rate (breaths per minute)	23.2 (0.2)	21.6 (0.3)	25.6 (0.4)	1.10 (1.08–1.13)	<0.001	22.9 (0.2)	24.6 (0.5)	1.03 (1.01–1.06)	0.002
SpO_2_	95.5 (0.1)	96.0 (0.1)	94.8 (0.2)	0.91 (0.88–0.95)	<0.001	95.6 (0.1)	95.0 (0.3)	0.96 (0.92–0.99)	0.021
FiO_2_	34.7 (0.7)	25.7 (0.4)	47.8 (1.3)	1.10 (1.08–1.11)	<0.001	31.9 (0.6)	45.8 (1.8)	1.03 (1.02–1.03)	<0.001
SpO_2_/FiO_2_ Ratio	340.3 (3.8)	398.7 (3.5)	259.2 (6.2)	0.99 (0.99–0.99)	<0.001	357.2 (4.0)	273.7 (8.7)	0.99 (0.99–1.00)	<0.001
**Race, No. (%)**									
White	438 (42.0)	219 (58.9)	153 (41.1)			321 (73.3)	117 (26.7)		
Black	187 (17.9)	110 (62.9)	65 (37.1)	0.85 (0.58–1.22)	0.375	150 (80.2)	37 (19.8)	0.68 (0.45–1.03)	0.067
Hispanic	113 (10.8)	70 (64.2)	39 (35.8)	0.80 (0.51–1.24)	0.316	96 (85.0)	17 (15.0)	0.49 (0.28–0.85)	0.011
Asian	37 (3.6)	15 (45.5)	18 (54.5)	1.72 (0.84–3.51)	0.138	33 (89.2)	4 (10.8)	0.33 (0.12–0.96)	0.042
Other/Mix	177 (17.0)	97 (55.4)	78 (44.6)	1.15 (0.80–1.65)	0.447	160 (90.4)	17 (9.6)	0.29 (0.17–0.50)	<0.001
Not recorded	90 (8.6)	36 (41.4)	51 (58.6)			72 (80.0)	18 (20.0)		
**Comorbidity, No. (%)**									
Diabetes	443 (42.5)	203 (37.1)	194 (48.0)	1.56 (1.20–2.03)	<0.001	332 (39.9)	111 (52.9)	1.71 (1.26–2.32)	<0.001
CAD	182 (17.5)	85 (15.5)	60 (14.9)	0.95 (0.66–1.35)	0.762	123 (14.8)	59 (28.1)	2.27 (1.58–3.23)	<0.001
Hypertension	588 (56.4)	294 (53.7)	225 (55.7)	1.08 (0.83–1.40)	0.572	446 (53.6)	142 (67.6)	1.83 (1.34–2.54)	<0.001
CKD	174 (16.7)	87 (15.9)	56 (13.9)	0.85 (0.59–1.22)	0.377	115 (13.8)	59 (28.1)	2.45 (1.70–3.50)	<0.001
COPD	123 (11.8)	54 (9.9)	44 (10.9)	1.11 (0.73–1.69)	0.616	78 (9.4)	45 (21.4)	2.65 (1.76–3.96)	<0.001
Cancer	166 (15.9)	89 (16.3)	47 (11.6)	0.67 (0.45–0.97)	0.038	113 (13.6)	53 (25.2)	2.14 (1.47–3.09)	<0.001
									
**Symptom, No. (%)**									
Dyspnea	739 (70.9)	340 (62.2)	342 (84.7)	3.37 (2.45–4.69)	<0.001	590 (70.9)	149 (71.0)	1.03 (0.74–1.45)	0.875
LOC	30 (2.9)	19 (3.5)	7 (1.7)	0.48 (0.19–1.12)	0.105	22 (2.6)	8 (3.8)	1.47 (0.61–3.22)	0.36
Hemoptysis	13 (1.2)	7 (1.3)	5 (1.2)	0.96 (0.28–3.02)	0.939	12 (1.4)	1 (0.5)	0.33 (0.02–1.68)	0.285
Smoking, No. (%*)									
Never	387 (37.1)	211 (57.7)	155 (42.3)			323 (83.5)	64 (16.5)		
Former	231 (22.2)	106 (53.5)	92 (46.5)	1.18 (0.83–1.67)	0.347	163 (70.6)	68 (29.4)	2.11 (1.43–3.11)	<0.001
Current	86 (8.3)	37 (47.4)	41 (52.6)	1.51 (0.92–2.46)	0.100	70 (81.4)	16 (18.6)	1.15 (0.63–2.11)	0.64
Not Recorded	338 (32.4)	193 (62.5)	116 (37.5)			276 (81.7)	62 (18.3)		
**Medication, No. (%)**									
Statin	511 (49.0)	269 (49.2)	180 (44.6)	0.84 (0.65–1.09)	0.182	389 (46.8)	122 (58.1)	1.63 (1.20–2.22)	0.002
RAAS inhibitor	315 (30.2)	162 (29.6)	126 (31.2)	1.08 (0.81–1.42)	0.602	248 (29.8)	67 (31.9)	1.10 (0.79–1.52)	0.554
Aspirin	318 (30.5)	170 (31.1)	106 (26.2)	0.80 (0.60–1.06)	0.122	232 (27.9)	86 (41.0)	1.83 (1.33–2.50)	<0.001
Anticoagulant	44 (4.2)	14 (2.6)	16 (4.0)	1.57 (0.76–3.25)	0.225	26 (3.1)	18 (8.6)	2.91 (1.56–5.41)	<0.001
**bnormal X-ray, No. (%)**	818 (78.5)	386 (70.6)	366 (90.6)	3.88 (2.62–5.87)	<0.001	644 (77.4)	174 (82.9)	1.42 (0.95–2.19)	0.1

Patients coded for “do not intubate” were excluded from analyses for ventilation status. P-values are for univariate logistic regression analyses. COPD = chronic obstructive pulmonary disorder; LOC = loss of consciousness; RAAS = renin angiotensin-aldosterone system; OR = odds ratio; CI = confidence interval; SD = standard deviation.

⁎ Calculated as a percentage of each group (race or smoking status). Otherwise, values were calculated as a percentage of each outcome group (ventilation or discharge). Odds ratios in the ethnicity and smoking categories were calculated relative to white patients or never smokers, respectively. ^∞^ Odds ratio calculated for every 10-year increase in age.

Among the 1042 patients admitted to hospital, 832 (79.8%) were discharged, and 210 (20.2%) died in hospital. 86% of patients who died had at least one comorbidity. As shown in **Supplemental Table 2**, the most common cause of mortality from COVID-19 was respiratory failure followed by sepsis with multi-organ failure. The median length of stay was 10 (IQR: 6–21, Range = 1–110) days. The median length of ICU stay and ventilation duration were 15 (IQR: 7–23, Range = 1–84) and 13 (IQR: 7–22, Range = 1–84) days, respectively. The median length of stay among those that died was 11 (IQR = 6–20; Range = 1–57) days. 449 patients were admitted to the ICU, of which 404 (90.0%) were mechanically ventilated. Ninety-one patients were identified as “do not intubate” (DNI) on admission. As shown in **Supplemental Table 1**, DNI patients were on average older and had a higher prevalence of pre-existing conditions than patients included in the final study. One hundred and thirty-six (33.7%) mechanically ventilated patients died.

### 3.2 Patient factors associated with severity of disease

In univariate analyses, clinical factors that were associated with need for mechanical ventilation and in-hospital mortality were identified (detailed in Table 1 and Table 2). Many variables on admission were consistently predictive of both ventilation need and in-hospital mortality, including male sex, diabetes mellitus, cancer, admission vital signs including diastolic blood pressure, respiratory rate, SpO_2_, and SpO_2_:FiO_2_ ratio, lower levels of albumin and eGFR, and elevated absolute neutrophils, anion gap, activated partial thromboplastin and prothrombin time, blood urea nitrogen (BUN), C-reactive protein (CRP), creatinine, d-dimer, eGFR, plasma glucose, neutrophil to lymphocyte ratio, procalcitonin, and troponin T (high sensitivity). Troponin predicted both need for mechanical ventilation and in-hospital mortality when assessed as a continuous variable or as a dichotomous variable with the threshold of >10 ng/L used to indicate the presence of myocardial injury [12]. However, it was striking that many factors were only associated with one of either mechanical ventilation need or in-hospital mortality. Those that met significance for ventilation need only included dyspnea, x-ray abnormality, heart rate, and systolic blood pressure on admission, and elevated alanine aminotransferase (ALT), aspartate aminotransferase (AST), direct bilirubin, erythrocyte sedimentation rate (ESR), ferritin, fibrinogen, lactate dehydrogenase (LDH), mean corpuscular hemoglobin (MCH), and white blood cell count (WBC). The variables that were predictive of mortality only included age, CAD, hypertension, CKD, COPD, statin, aspirin, and anticoagulant use, lower levels of hemoglobin and platelets, and elevated levels of creatinine, mean corpuscular volume (MCV), NT-ProBNP, and increased red cell distribution and width (RDW).

**Table 2:** Laboratory results of hospitalized COVID-19 patients on admission.

		Ventilation Status			Mortality		
Lab test	Mean (SEM) Total	Mean (SEM) Not ventilated (*n* = 550)	Mean (SEM) Ventilated (*n* = 401)	P-Value	Mean (SEM) Discharged (*n* = 829)	Mean (SEM) Deceased (*n* = 211)	P-Value
Absolute Lymphocytes (K/μL)	1.36 (0.17)	1.38 (0.23)	1.35 (0.29)	0.945	1.31 (0.16)	1.56 (0.52)	0.569
Absolute Neutrophils (K/μL)	5.98 (0.12)	5.28 (0.17)	6.91 (0.19)	<0.001	5.81 (0.14)	6.66 (0.30)	0.01
Albumin (g/dL)	3.62 (0.02)	3.74 (0.02)	3.46 (0.03)	<0.001	3.67 (0.02)	3.38 (0.04)	<0.001
Anion Gap (mmol/L)	16.28 (0.11)	15.92 (0.15)	16.85 (0.19)	<0.001	16.15 (0.12)	16.81 (0.29)	0.019
Alanine aminotransferase (U/L)	52.52 (7.41)	36.31 (1.54)	78.15 (18.70)	<0.001	47.86 (3.92)	70.92 (33.30)	0.28
aPTT (s)	36.84 (0.66)	34.10 (0.54)	39.01 (1.16)	0.002	35.87 (0.64)	40.42 (1.95)	0.009
Aspartate aminotransferase (U/L)	79.51 (15.52)	46.75 (1.93)	130.5 (39.64)	<0.001	65.62 (7.64)	134.5 (70.78)	0.194
Blood Urea Nitrogen (mg/dL)	22.19 (0.58)	19.76 (0.72)	23.89 (1.03)	<0.001	19.35 (0.56)	33.43 (1.61)	<0.001
C-reactive protein (mg/L)	109.6 (2.84)	79.85 (3.13)	154.3 (4.88)	<0.001	102.8 (3.11)	136.2 (6.53)	<0.001
Creatinine (mg/dL)	1.48 (0.06)	1.35 (0.07)	1.61 (0.11)	0.045	1.35 (0.06)	1.99 (0.14)	<0.001
Creatine Kinase (U/L)	764.0 (364.2)	976.9 (690.4)	586.4 (131.3)	0.644	794.3 (455.0)	645.0 (163.3)	0.87
D-dimer (ng/L)	1905 (108.7)	1494 (69.06)	2370 (251.4)	<0.001	1757 (128.7)	2495 (169.5)	0.047
Direct bilirubin (mg/dL)	0.22 (0.01)	0.17 (0.02)	0.28 (0.03)	<0.001	0.21 (0.02)	0.25 (0.03)	0.246
eGFR (mL/min/1.73m^2^)	67.89 (0.93)	71.51 (1.24)	66.63 (1.53)	0.013	72.68 (0.99)	48.96 (1.90)	<0.001
ESR (mm/hr)	50.33 (1.13)	47.83 (1.51)	54.77 (1.90)	0.004	49.67 (1.24)	52.92 (2.75)	0.251
Ferritin (μg/mL)	1283 (128.0)	813.9 (51.38)	2029 (315.6)	<0.001	1183 (130.7)	1690 (371.5)	0.144
Fibrinogen (mg/dL)	578.3 (8.41)	531.5 (10.12)	634.6 (13.45)	<0.001	583.3 (9.44)	556.7 (18.41)	0.217
Glucose (mg/dL)	157.4 (2.94)	147.7 (4.10)	172.5 (4.84)	<0.001	152.5 (3.18)	177.0 (7.20)	0.001
Hemoglobin (g/dl)	12.86 (0.07)	12.86 (0.09)	13.03 (0.11)	0.216	13.00 (0.07)	12.29 (0.17)	<0.001
Lactate Dehydrogenase (U/L)	408.4 (18.50)	323.3 (7.11)	530.1 (43.96)	<0.001	384.9 (11.45)	503.4 (80.72)	0.105
MCH (pg/cell)	28.96 (0.08)	28.80 (0.11)	29.19 (0.11)	0.017	28.88 (0.09)	29.25 (0.17)	0.052
MCV (fL/cell)	87.86 (0.20)	87.35 (0.29)	88.12 (0.31)	0.071	87.35 (0.22)	89.86 (0.48)	<0.001
Neut:Lymph Ratio	8.92 (0.41)	6.75 (0.38)	11.48 (0.82)	<0.001	7.67 (0.34)	13.87 (1.48)	<0.001
NT_Pro-BNP (pg/mL)	3233 (516.6)	2664 (810.2)	3479 (716.2)	0.472	2328 (587.8)	6030 (1048)	0.009
Platelets (×10^9^/L)	214.5 (2.97)	214.0 (4.02)	216.7 (4.97)	0.673	220.5 (3.29)	190.7 (6.61)	<0.001
Procalcitonin (ng/mL)	1.41 (0.26)	0.63 (0.19)	2.28 (0.53)	0.018	0.81 (0.19)	3.74 (1.02)	0.001
Prothrombin Time (s)	15.10 (0.22)	14.42 (0.16)	15.50 (0.43)	0.009	14.67 (0.15)	16.62 (0.83)	0.001
RDW (%)	14.09 (0.06)	14.09 (0.09)	13.88 (0.08)	0.11	13.90 (0.07)	14.85 (0.13)	<0.001
Troponin T (ng/L)	45.60 (3.85)	32.04 (4.12)	55.31 (7.10)	0.008	35.16 (3.81)	87.08 (11.25)	<0.001
WBC (×10^9^/L)	7.97 (0.22)	7.22 (0.29)	8.87 (0.36)	<0.001	7.74 (0.22)	8.86 (0.62)	0.068

Patients coded for “do not intubate” were excluded from analyses for ventilation status. P-values are for univariate logistic regression analyses. aPTT = activated partial thromboplastin time; eGFR = estimated glomerular filtration rate; ESR = erythrocyte sedimentation rate; MCH = mean corpuscular hemoglobin; MCV = mean corpuscular volume; RDW=red cell distribution width; WBC = white blood cell; SD = standard deviation.

**Fig. 1:**
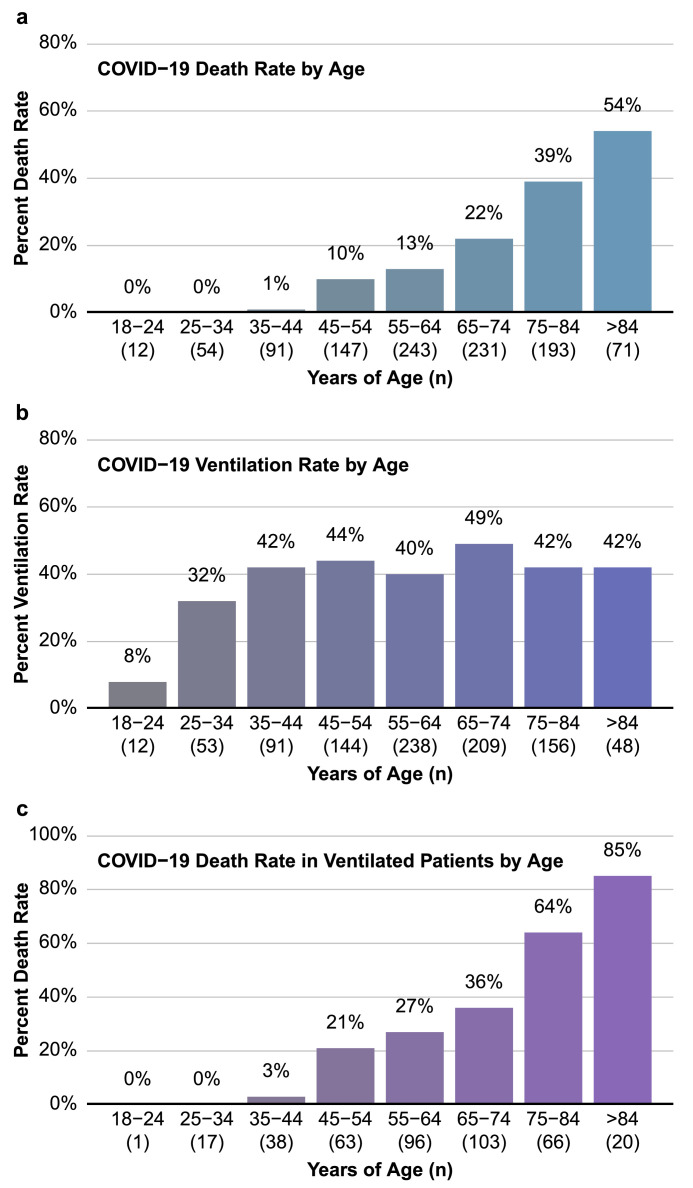
Mortality rate, but not need for mechanical ventilation, increases with age. Mortality rate (a), ventilation rate (b) and mortality rate in ventilated patients (c) were plotted against decade of life.

**Fig. 2:**
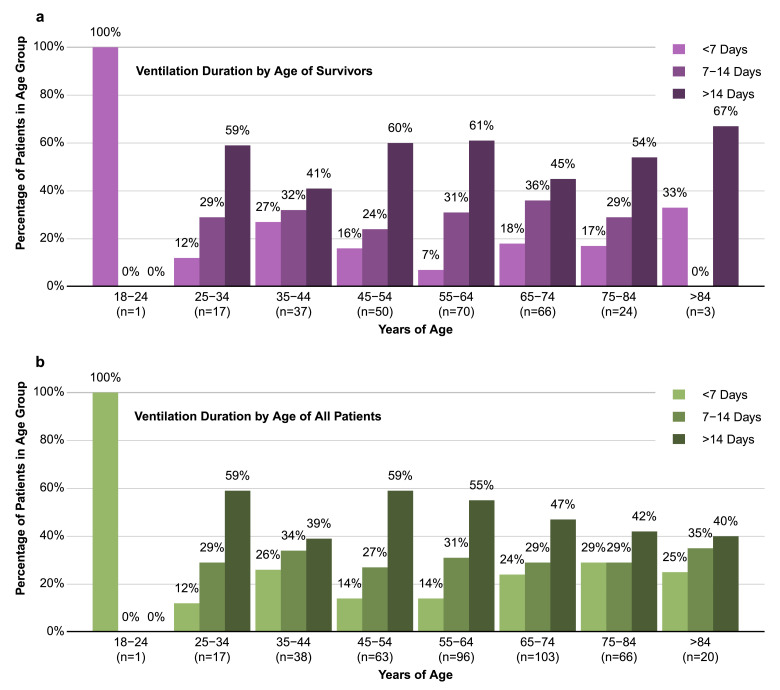
Young people hospitalized with COVID-19 are equally at risk of long ventilation periods as elderly patients. Proportion of length of mechanical ventilation (<7, 7–14, and >14 days) requirement stratified by different age groups in survivors (a) and in the entire cohort (b). .

It was notable that age was not a significant predictor of whether a patient would require mechanical ventilation (*p* = 0.12). To investigate this further, we determined rates of mortality and need for mechanical ventilation per decade of life. As anticipated, older age was associated with an increase in mortality rate (Fig. 1**a**). However, other than patients in the youngest age groups, the percentage of hospitalized COVID-19 patients requiring mechanical ventilation was similar in each decade of life (Fig. 1**b**). Of those that were ventilated, there is a clear correlation between age and risk of in-hospital death. Indeed, of patients in the oldest group (>84 years of age), only 15% survived if mechanical ventilation was required (Fig. 1**c**). Interestingly, young patients were as likely as patients of advanced age to require long durations of mechanical ventilation (Fig. 2). In fact, 78% of ventilated patients between ages 18 and 44 were intubated for longer than 6 days, and 45% were intubated for longer than 14 days. It should be noted that patients who were DNI on admission had a median age of 78 years (IQR: 72–85, Range = 32–99), potentially explaining why patients in the oldest groups were less represented in the longer ventilation durations. However, the data clearly points to a need for long durations of ventilation in COVID-19 patients, including younger age groups.

### 3.3 Multivariable logistic regression models to predict mechanical ventilation and in-hospital mortality

Multivariable logistic regression models were used in order to develop risk scores to predict important clinical outcomes in COVID-19, namely the need for mechanical ventilation and in-hospital mortality. As detailed in the above section, we found many variables, including age, that were distinctly associated with either the need for mechanical ventilation or for in-hospital mortality, but not for both. We therefore constructed separate risk scores for ventilation requirement (VICE=Ventilation In COVID Estimate) and in-hospital death (DICE=Death In COVID Estimate) based on multivariable logistic regression models. We divided our overall study into separate derivation (MGH, *n* = 578) and independent validation (BWH, NWH, BWFH, NSMC; *n* = 464) cohorts based on the hospital of admission. Our derivation cohort of 578 patients (**Supplemental Table 3**) had a median age of 62 years [IQR, 51–73], consisted of 346 (59.9%) males, and 111 (19.2%) died in the hospital. Excluding patients choosing a DNI status, 243 (46.1%) required mechanical ventilation. Similar to the overall cohort, hypertension (*n* = 304; 52.6%) and diabetes (*n* = 254; 43.9%) were the most common comorbidities.

Using a stepwise backwards regression approach, 4 variables were independently associated with the need for mechanical ventilation (Table 3). These variables included diabetes mellitus (OR 2.11; 95% CI 1.34–3.34, *p* = 0.001), SpO_2_:FiO_2_ Ratio (for every 100 increase, OR 0.42; 95% CI 0.34–0.53, *p*<0.001), CRP (log_2_-transformed value, OR 1.33; 95% CI 1.12–1.57, *p* = 0.001), and LDH (log_2_-transformed value, OR 2.08; 95% CI 1.34–3.23, *p* = 0.001).

**Table 3:** Multivariable logistic regression models for predicting mechanical ventilation need (VICE) and mortality (DICE) in COVID-19 patients.

Variable	Odds Ratio (95% CI)	P-Value
**Mechanical Ventilation**		
Diabetes mellitus	2.114 (1.340–3.337)	0.001
SpO_2_:FiO_2_ Ratio (for every 100 increase)	0.423 (0.336–0.532)	<0.001
C-Reactive Protein‡ (mg/L)	1.328 (1.120–1.574)	0.001
Lactate Dehydrogenase‡ (U/L)	2.083 (1.341–3.234)	0.001
**Mortality**		
Age (for every 10 years)	2.953 (2.227–3.916)	<0.001
Male Sex	3.026 (1.534–5.969)	0.001
Coronary Artery Disease	2.792 (1.351–5.770)	0.006
Diabetes mellitus	2.159 (1.175–3.967)	0.013
Statin (chronic use)	0.467 (0.237–0.920)	0.028
SpO_2_:FiO_2_ Ratio (for every 100 increase)	0.475 (0.362–0.622)	<0.001
Body Mass Index	1.067 (1.017–1.120)	0.008
Neut:Lymph Ratio (for 10x increase)	1.323 (1.001–1.441)	0.045
Platelets (for every 50×10^9^/L increase)	0.775 (0.635–0.947)	0.013
Procalcitonin‡ (ng/mL)	1.238 (1.064–1.441)	0.006

The constant value in the ventilation regression analysis was β_0_ = −5.62.

The constant value in the mortality regression analysis was β_0_ = −8.26.

‡ Log base 2 transformed value.

We identified 10 variables (Table 3) that independently associated with the odds of in-hospital death including: age (for every 10 year increase: OR 2.95; 95% CI 2.23–3.92, *p*<0.001), male sex (OR 3.03; 95% CI 1.53–5.97, *p* = 0.001), CAD (OR 2.79; 95% CI 1.35–5.77, *p* = 0.006), diabetes mellitus (OR 2.16; 95% CI 1.18–3.97, *p* = 0.013), chronic statin use (OR 0.47; 95% CI 0.24–0.92, *p* = 0.028), SpO_2_:FiO_2_ ratio (for every 100 increase, OR 0.48; 95% CI 0.36–0.62, *p*<0.001), BMI (OR 1.07; 95% CI 1.02–1.12, *p* = 0.008), neutrophil to lymphocyte ratio (OR 1.32 for every 10 unit increase; 95% CI 1.00–1.44, *p* = 0.045), platelet count (OR 0.78 for every 50 × 10^9^/L increase; 95% CI 0.64–0.95, *p* = 0.013), and procalcitonin (log_2_-transformed, OR 1.24; 95% CI 1.06–1.44, *P* = 0.006). Of note, use of an angiotensin converting enzyme inhibitor (ACEI) or angiotensin receptor blocker (ARB) was not associated with a difference in outcome both in univariate and multivariable analysis.

### 3.4 Performance of the VICE and DICE risk scores

**Fig. 3:**
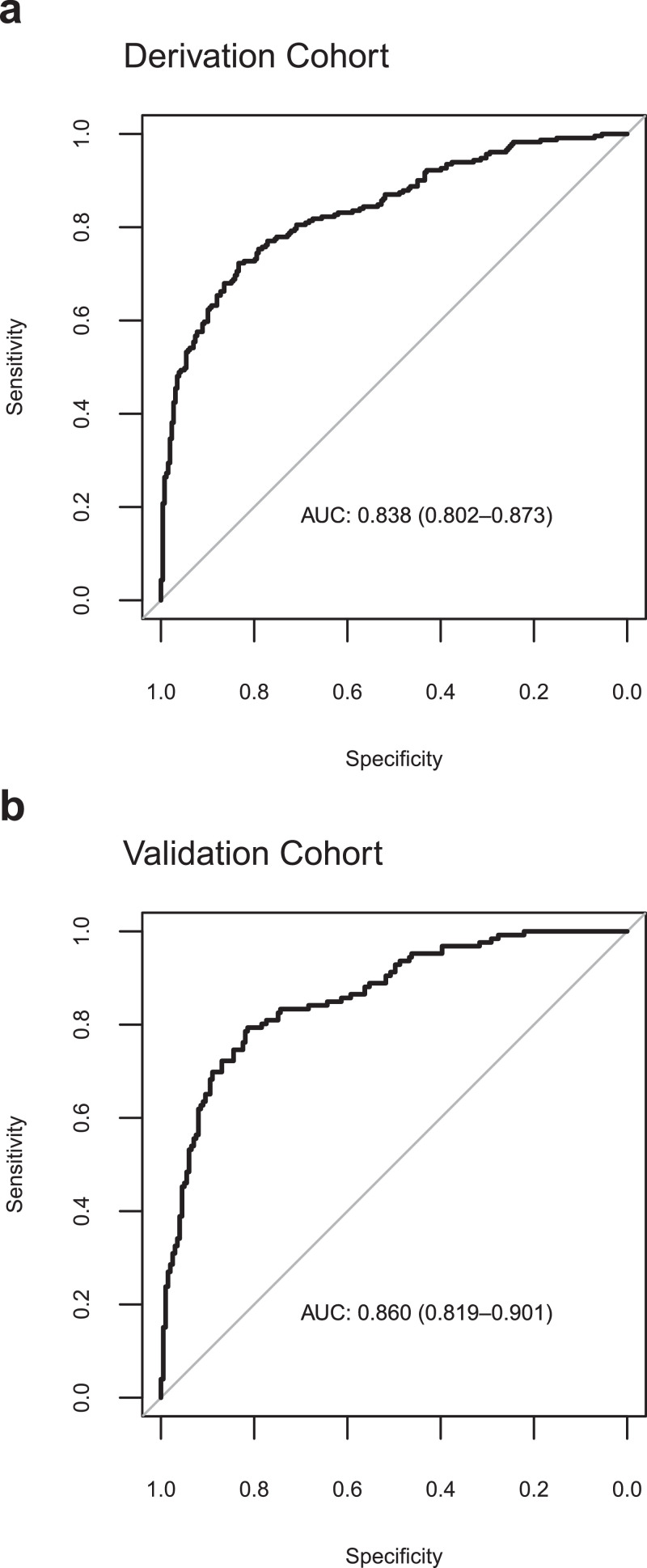
Performance of the VICE score. The receiver-operator characteristic (ROC) curve (with area under the curve, AUC) of predicting mechanical ventilation requirements among patients with COVID-19 in the derivation cohort (a) and validation cohort (b) using the VICE score.

**Fig. 4:**
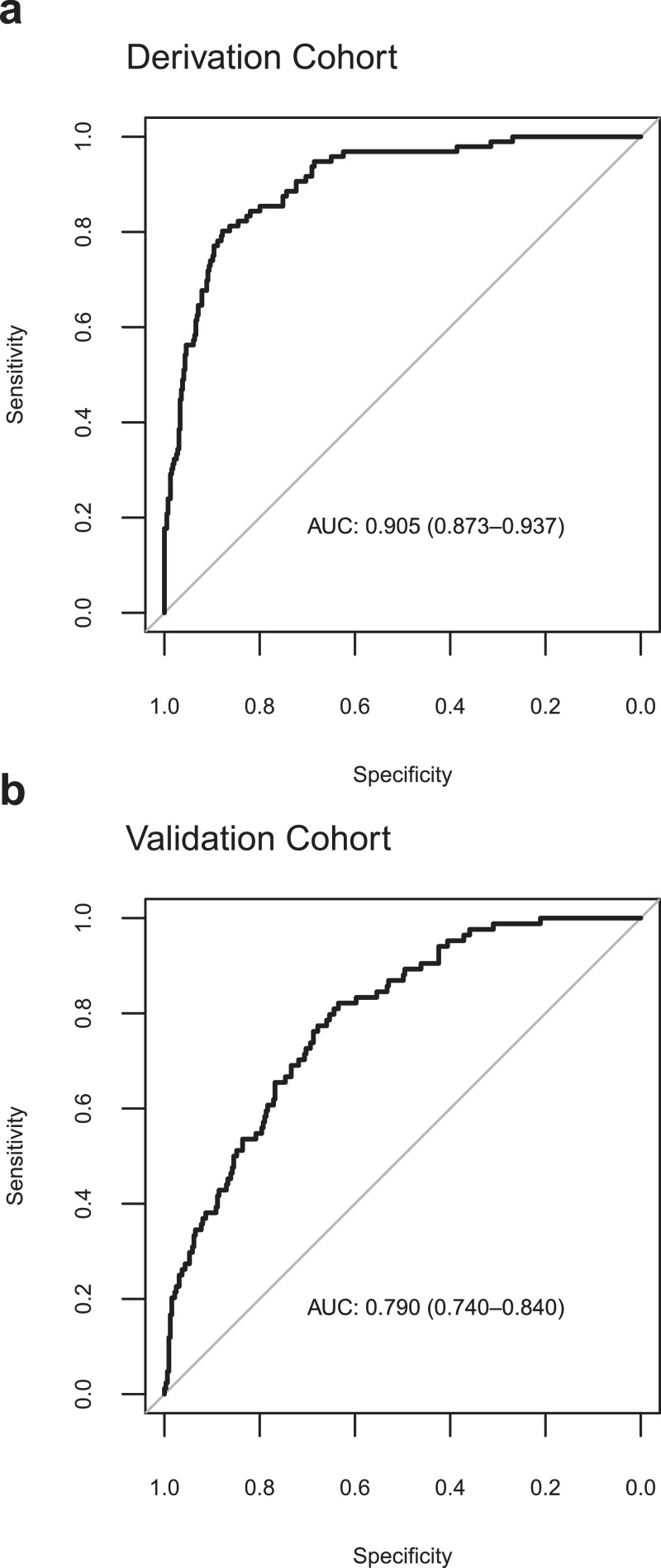
Performance of the DICE score. The receiver-operator characteristic (ROC) curve (with area under the curve, AUC) of predicting in-hospital mortality among patients with COVID-19 in the derivation cohort (a) and validation cohort (b) using the DICE score.

**Fig. 5:**
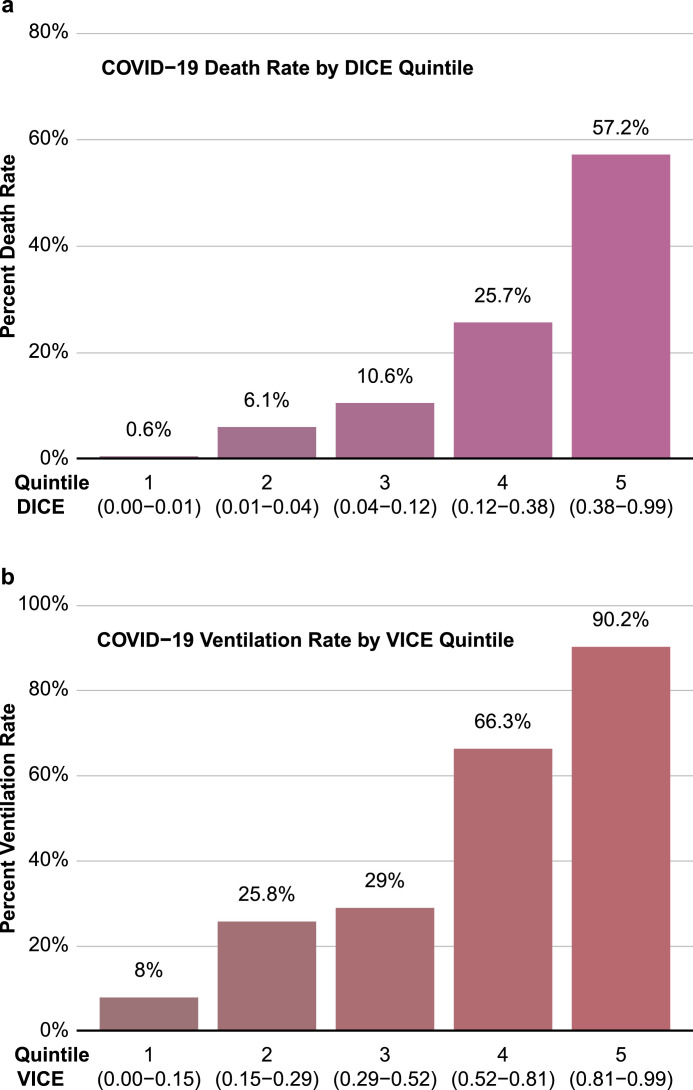
Performance of the VICE and DICE scores in the entire cohort of patients. Ventilation rate was plotted against VICE score quintiles (a). Mortality rate was plotted against DICE quintiles (b).

The VICE and DICE risk scores were constructed based on coefficients from the multivariate logistic regression models. We used the following formula to calculate the probability (p): $p=\frac{e^{\left(\beta_{0}+\beta_{1}X_{1}+\beta_{2}X_{2}+\ldots \right)}}{1+e^{\left(\beta_{0}+\beta_{1}X_{1}+\beta_{2}X_{2}+\ldots \right)}}$  where β=ln(OR) and $\beta_{0}$ can be found in the caption to Table 3. An online calculator based on the VICE and DICE risk scores is available freely, allowing health care providers to enter values for the variables required to calculate the risk for mechanical ventilation and in-hospital mortality at https://covid-calculator.com/. In receiver operator characteristic (ROC) curve analysis, the area under the curve (AUC or C-statistic) of the VICE risk score in the derivation cohort was 0.84 (Fig. 3**a**, 95% CI, 0.80–0.87), and 0.86 (Fig. 3**b**, 95% CI, 0.82–0.90) in the validation cohort. The DICE risk score for in-hospital mortality in the derivation cohort had an AUC of 0.91 (Fig. 4**a**, 95% CI, 0.87–0.94). The AUC in the validation cohort was 0.79 (95% CI 0.74–0.84) (Fig. 4**b).** As shown in Fig. 5, progressive increases in ventilation (Fig. 5**a**) and in-hospital mortality (Fig. 5**b**) rates were observed with increasing quintiles of VICE and DICE scores, respectively. In patients falling within the highest quintile of the DICE score, mortality was 58% compared to 0.6% in the lowest quintile.

**Supplemental Table 4** describes the racial demographics and baseline characteristics in the derivation and validation cohorts. Although the proportion of patients from white and Hispanic backgrounds are similar, the proportion of patients from a Black background are more than doubled in the validation cohort. In addition, the validation cohort was older and less represented by males than the derivation cohort. Despite these differences, the VICE and DICE risk scores demonstrated robust accuracy for predicting the need for mechanical ventilation and in-hospital mortality in both cohorts.

Previous reports have highlighted a marked increase in risk of developing severe illness in COVID-19 in minority ethnic groups [[13], [14], [15], [16]]. We found it interesting, therefore, that we did not observe this in our univariate analyses (Table 1), with the data even pointing to worse mortality rates in white patients. We investigated if patients from white backgrounds were in poorer health on admission to hospital by analyzing their DICE scores. Indeed, we observed that white patients in our cohort were at significantly higher risk of in-hospital death on admission by the DICE score than Black (*p* = 0.0006) or Hispanic (*p*<0.0001) patients (**Supplemental Figure 1**, Median DICE Score [IQR] in: White = 0.13 [0.04–0.42)]; Black = 0.08 [0.01–0.24]; Hispanic = 0.04 [0.01–0.16]). Patients from an Asian background also presented with lower risk (Asian = 0.11 [0.03–0.20]) compared to white patients, although this did not meet statistical significance (*p* = 0.287). After adjusting for DICE score, race was no longer a predictor of in-hospital mortality. We also considered whether there were a disproportionate number of minority patients in the DNI and/or CMO groups that were excluded from our analyses, therefore explaining the discordant results with the published literature. As shown in **Supplemental Table 1**, this does not appear to be explained by the CMO population, which actually had a higher percentage of white patients than any other background.

Liang et al. recently developed and validated a clinical risk score to predict the development of severe illness (combined end-points of ICU admission, ventilation requirement and death) among hospitalized COVID-19 patients [7]. The algorithm performed very well in cohorts from Hubei (AUC = 0.87) and outside Hubei (AUC = 0.82), but we do not know how well it performs in other populations, including the US. The accuracy of the COVID-GRAM risk score at predicting the combined endpoint of ICU admission, mechanical ventilation requirement, and in-hospital death in our derivation cohort was 0.71 (0.67–0.76) and 0.70 (0.64–0.75) in our validation cohort (**Supplemental Figure 2**). The lower AUC for COVID-GRAM in our population may be due to geographical differences in COVID-19 presentation and outcomes in China compared to Boston. In addition, we assessed the accuracy of a modified SOFA score, a well-established model to predict mortality of critically ill patients, to predict in-hospital death due to COVID-19 [17,18]. As shown in **Supplemental Figure 3**, the modified SOFA score (AUC = 0.70, 95% CI, 0.66–0.74) was less accurate at predicting in-hospital mortality in COVID-19 patients in comparison to the DICE score (AUC = 0.85, 95% CI, 0.82–0.88, *p*<0.0001) in our entire cohort.

## 4 Discussion

This study reports on the in-hospital outcomes of sequentially hospitalized patients with COVID-19 in the Boston area. We identified many independent risk factors for mortality in this population, including older age, male sex, preexisting diabetes mellitus and CAD, lower SpO_2_:FiO_2_ ratio, increased BMI, thrombocytopenia, and higher levels of the inflammatory and infectious biomarkers procalcitonin, and neutrophil to lymphocyte ratio. Interestingly, we also found that chronic statin use was associated with a lower risk of in-hospital death, perhaps supporting the anti-inflammatory and immunomodulatory benefits of statins in this disease [[19], [20], [21]].

Notably, only preexisting diabetes and SpO_2_:FiO_2_ ratio were included in both risk score models. Factors that uniquely and independently predicted ventilation requirement included elevated CRP and LDH. Age was not a significant predictor of ventilation need, perhaps dispelling the belief that COVID-19 only severely affects the elderly. It has been suggested that people under 40 are increasingly driving the spread of COVID-19 [22]. While there is clear evidence that young patients are less likely to die from COVID-19, young hospitalized patients regularly require the use of ventilators for extended periods of time, thus stressing a system that is already in short supply of ICU beds. Recognizing the difference in variables that independently predict need for ventilation and death in COVID-19 is greatly important, especially as current risk score calculators combine ICU admission, ventilation needs and mortality into one endpoint [7]. The use of two independent risk scores predicting ventilation need and in-hospital death would allow health care systems to not only more precisely prognosticate individual patient outcomes but also better predict demand for mechanical ventilators and ICU beds.

In our study, we demonstrated that excessive levels of inflammatory and infectious markers, such as CRP, LDH and procalcitonin, were associated with worse outcomes. Given the relationship between cardiometabolic disease and death in COVID-19, it is possible that these patients are more vulnerable to the aggressive inflammatory response induced by the virus [23]. Of note, it has recently been suggested that biological, rather than chronological, aging is a stronger predictor of all-cause mortality related to COVID-19 [24]. Therefore, underlying age-related cardiovascular dysfunction may increase the risk of a hyperinflammatory response that augments the effects of COVID-19 in these individuals [23,25,26]. Interestingly, chronic statin use was associated with reduced in-hospital mortality, further underscoring the strong link between underlying cardiovascular disease and worse outcomes with COVID-19 [8,[27], [28], [29], [30], [31], [32], [33], [34], [35]].

Importantly, we developed two novel prediction models to calculate risk of hospitalized COVID-19 patients developing severe outcomes. These models using clinical characteristics and laboratory assessment at the time of admission were effective at predicting risk of ventilation need and in-hospital death in both our derivation and validation cohorts. Of note, the two cohorts showed considerable variability in several factors, including sex and age, demonstrating our risk scores may be accurate in distinct populations from the United States. We also observed that the factors used to construct each risk score were different, demonstrating that it is beneficial to consider risks of ventilation need and in-hospital mortality separately. Interestingly, and in contrast to previous reports, we did not observe worse outcomes in COVID-19 patients from minority ethnic backgrounds [[13], [14], [15], [16]]. However, some caveats are worthy of mention. For example, socioeconomic status was not assessed in our study, which could be another important determinant of outcome [16]. Indeed, the patients included were inpatients only, and inequitable access to health care (including hospital admission) appears to contribute to the disproportionate impact of COVID-19 on patients from racial minorities in the United States. [36]. It has even been suggested that factors associated with low socioeconomic status, such as overcrowded housing and lack of opportunities to work from home, are more important in determining risk than racial background [37]. The data from our study was unable to quantify these risk factors. In addition, the proportion of Black patients admitted to the hospitals in our cohorts seems to fall lower than the most recent U.S. Census Bureau statistics for the Boston area [38]. Since we were only able to obtain data from the Mass General Brigham hospital network, it is possible that this population is not fully representative of the Boston-wide population.

The variables that we found to predict mechanical ventilation requirement and mortality are either readily available or routinely measured upon admission to the hospital. We anticipate that clinicians will easily be able to implement the DICE and VICE scores to stratify risk in admitted patients. In situations where hospital resources are plentiful, clinicians could use both scores to identify which patients are most likely to develop severe illness, and plan accordingly to monitor higher risk patients more intensely. However, under the most critical of circumstances where ventilators are in short supply, clinicians may require aid in triage and ventilator utilization. For example, ventilators may be prioritized for those patients who are at most risk for ventilation need (high VICE score) while still having a relatively lower risk of death (assessed by the DICE score).

One of the main strengths of the study was our ability to follow all patients from admission to the primary endpoint of either discharge or in-hospital death. A large fraction of patients (18%) remained in the hospital for longer than 28 days, indicating that analysis of full in-hospital outcomes (rather than assessing 28-day outcomes) provides a more complete risk stratification of COVID-19 patients. In addition, data were obtained by detailed medical record review rather than reliance on billing codes. Given the different variables that predict ventilation needs and mortality, we believe another strength was the construction of distinct risk scores. One potential limitation is the modest sample sizes in both our derivation and validation cohorts. Despite the considerable differences in demographics and laboratory values between the two cohorts, both were from the same hospital system and generalizability of our findings to a broader population may be limited. Although the exclusion of CMO patients was justified in the current study, this may have resulted in underestimating the impact of malignancy on inpatient mortality. Further, we aimed to focus on admission findings in determining outcomes and hence our risk scores do not include the effects of different treatment regimens or specific factors related to the course of a patient's hospitalization (e.g., changes in hemodynamic or respiratory status during the hospitalization), which may further enhance the ability to predict in-hospital mortality. However, we believe that a risk score based on factors known at the time of admission will be valuable as a risk stratification tool to health care providers caring for COVID-19 patients. Finally, although the DICE and VICE scores were validated in an independent cohort, a prospective study will be needed to further assess the validity of these risk scores.

This study identified baseline patient characteristics and admission laboratory values that associate with critical illness and in-hospital death in patients with COVID-19. In this investigation, we developed and validated risk score calculators to predict mechanical ventilation need and in-hospital death in COVID-19. These risk scores could potentially aid clinicians to better stratify risk in COVID-19 patients and optimize patient care and resource utilization in the surge of infections we are facing worldwide.

## Contributors

CJN and RM conceived, designed, and planned the study with input from LW and HHS. WT, WJ, RHL, HHS, and LW extracted the data from RPDR and EPIC. LW and HHS designed the code that was used to extract data from RPDR and analyze the data. CJN, HHS and LW performed the data analysis and designed the figures. CJN, LW and RM interpreted the data. CJN and RM drafted the manuscript with critical input from CLLC, LW, HHS and RHL.

All authors interpreted the data and made significant contributions to manuscript editing.

## Funding and Acknowledgments

Drs. Nicholson and Malhotra are supported by a COVID-19 Fast Grant (fastgrants.org). Dr. Malhotra is supported by NHLBI R01 HL142809, the American Heart Association grant 18TPA34230025, and the Wild Family Foundation. Dr. Lino Cardenas is supported by the MGH Physician-Scientist Development Award and the MGH Department of Medicine Pilot Translational Research Grant. We would like to thank Mr. Rohan Bosworth for creating the interactive website implementing the risk calculators (https://covid-calculator.com/).

## Data Sharing Statement

Summary data are available upon request of the corresponding authors.

## Declaration of Competing Interests

All authors have nothing to disclose.

## Appendix Supplementary materials

Image, application 1
