# Supplementary material for: Estimating risk of mechanical ventilation and in-hospital mortality among adult COVID-19 patients admitted to Mass General Brigham: The VICE and DICE scores
Source: eClinicalMedicine. 2021 Feb 25;33:100765. doi: 10.1016/j.eclinm.2021.100765 (PMC7906522; doi:10.1016/j.eclinm.2021.100765)
Supplement: Supplementary file 1 [file mmc1.pdf]

Supplemental Material

Supplemental Figure 1: DICE scores separated by ethnic group. DICE scores are plotted for patients with a White, Black, Hispanic or Asian ethnicity. Groups were compared using the Mann Whitney test.

Supplemental Figure 2: The area under the receiver-operator characteristic (ROC) curve (AUC) of COVID-GRAM predicting ICU admission, mechanical ventilation requirement, or death in the derivation (a) and validation (b) cohorts.

Supplemental Figure 3: The area under the receiver-operator characteristic (ROC) curve (AUC) of modified SOFA score (black text) vs. DICE (orange text) in predicting death in the entire cohort.

Supplemental Table 1: Baseline and admission characteristics of patients who were either DNI or CMO at the time of admission.

Supplemental Table 2: Causes of death in patients with COVID-19.

Supplemental Table 3: Baseline and admission characteristics of patients within the derivation cohort.

Supplemental Table 4: Comparison of baseline and admission characteristics of patients within the derivation and validation cohorts.

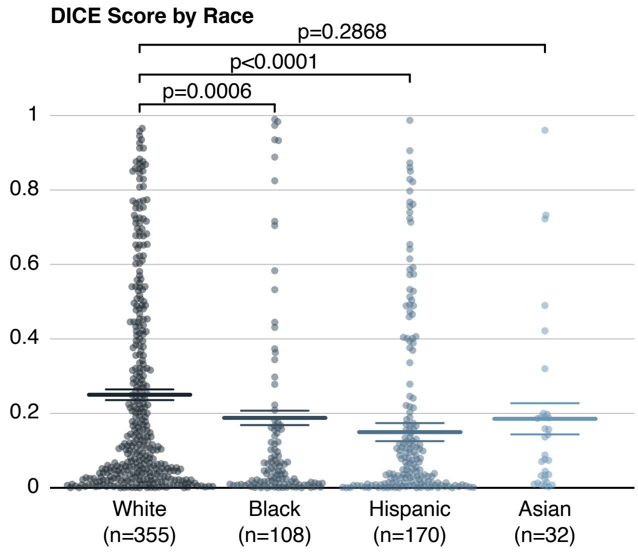

Supplemental Figure 1

a Derivation cohort

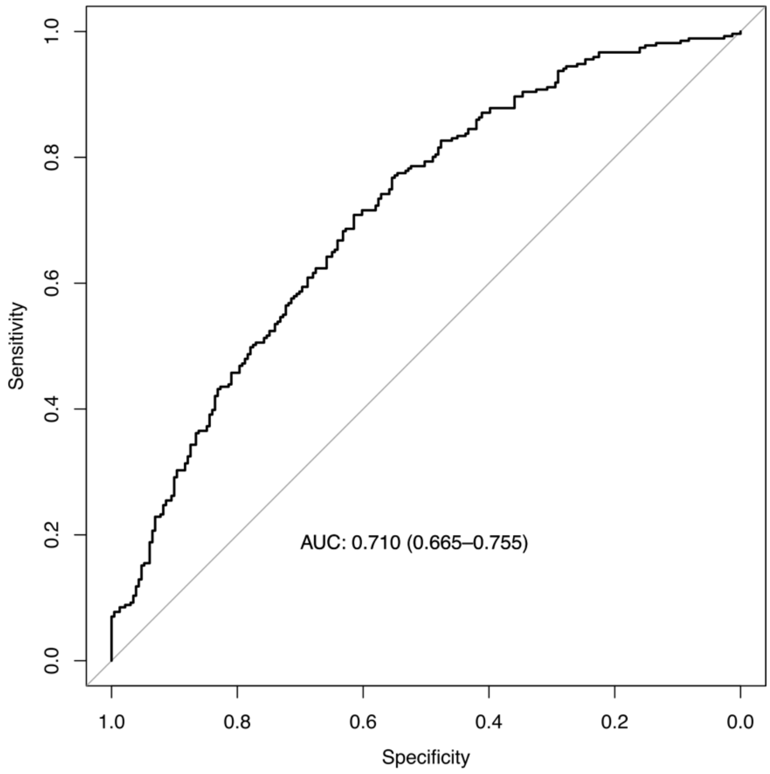

b Validation cohort

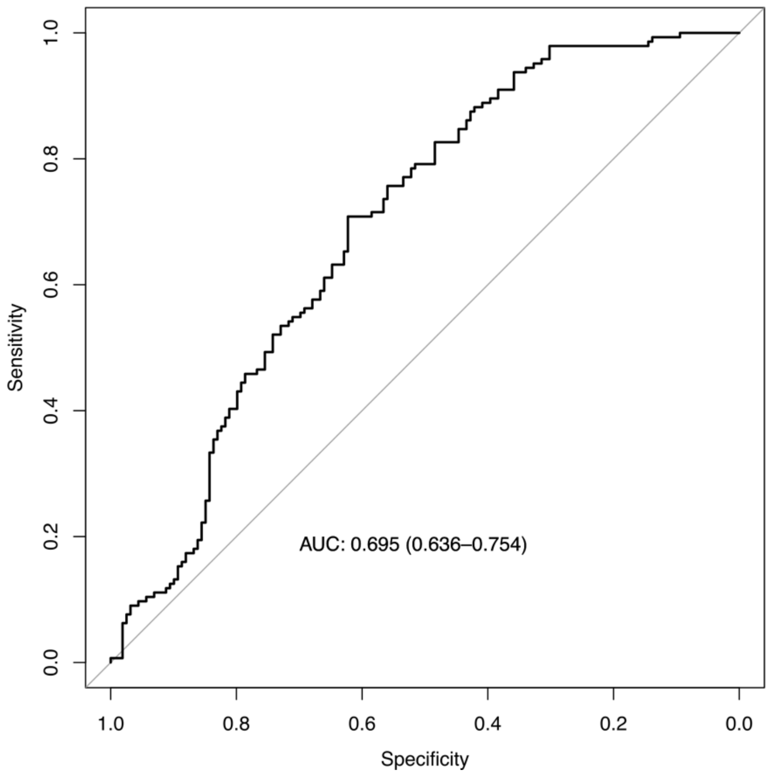

Supplemental Figure 2

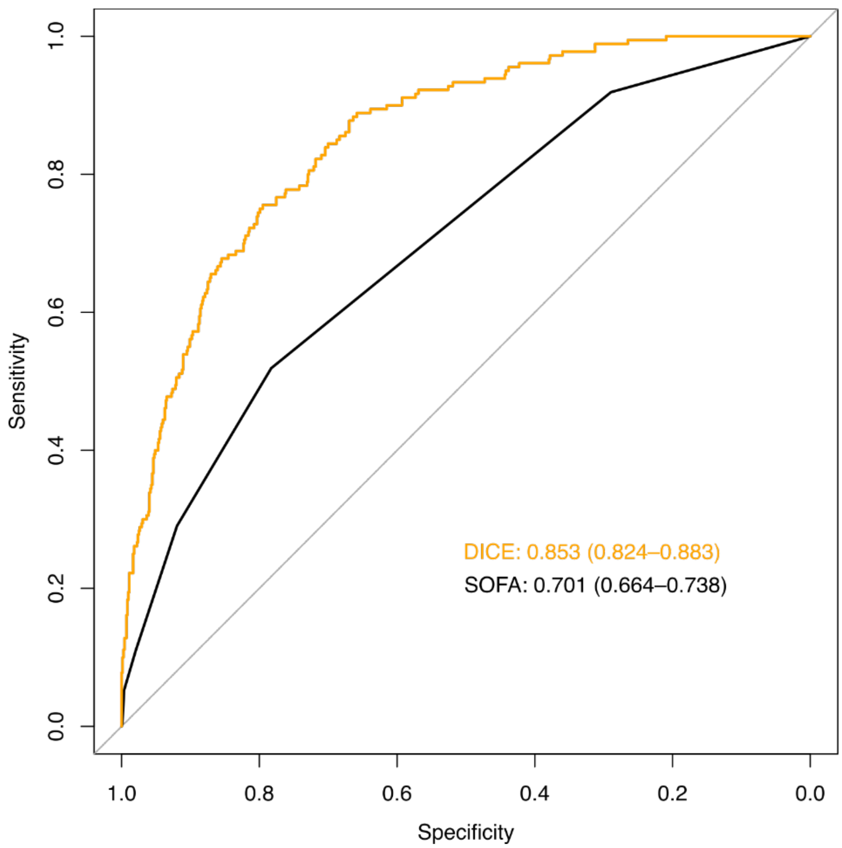

Supplemental Figure 3

Supplemental Table 1. Baseline and admission characteristics of patients who were either DNI or CMO at the time of admission.

Characteristic	All patients excluding CMO	Deceased	Excluded due to DNI status	Excluded due to CMO
Total patients	1042	210	91	95
Median (IQR) Age (years)	64 (53-75)	76 (66-82)	78 (72-85)	84 (77-91)
Male Sex (%)	592 (56.8)	139 (66.2)	44 (48.4)	44 (46.3)
**Race, No. (%*)**				
White	438 (42.0)	117 (26.7)	66 (15.1)	63 (12.6)
Black	187 (17.9)	37 (19.8)	12 (6.4)	14 (7.0)
Hispanic	113 (10.8)	17 (15.0)	4 (3.5)	4 (3.4)
Asian	37 (3.6)	4 (10.8)	4 (10.8)	4 (9.8)
Other/Mix	177 (17.0)	17 (9.6)	2 (1.1)	6 (3.3)
Not recorded	90 (8.6)	18 (20.0)	3 (3.3)	4 (4.3)
**Comorbidity, No. (%)**				
Diabetes	443 (42.5)	111 (52.9)	46 (50.5)	31 (32.6)
Coronary artery disease	182 (17.5)	59 (28.1)	37 (40.7)	35 (36.8)
Hypertension	588 (56.4)	142 (67.6)	69 (75.8)	71 (74.7)
Chronic kidney disease	174 (16.7)	59 (28.1)	31 (34.1)	27 (28.4)
COPD	123 (11.8)	45 (21.4)	25 (27.5)	23 (24.2)
Cancer	166 (15.9)	53 (25.2)	30 (33.0)	93 (97.9)

Supplemental Table 2. Causes of death in patients with COVID-19.

Causes of Mortality	No. of patients (%)
Respiratory	168 (80.0)
Sepsis/MOF	20 (9.5)
Respiratory and Sepsis/MOF	8 (3.8)
Cardiac	7 (3.3)
Renal Failure	3 (1.4)
Respiratory and Renal Failure	2 (1)
Cerebral Hemorrhage	1 (0.5)
Renal Failure and Encephalopathy	1 (0.5)

MOF= multi-organ failure; Cardiac includes cardiogenic shock, myocardial infarction, and cardiac arrest.

Supplement Table 3. Baseline Characteristics of the MGH Cohort Stratified by Need for Mechanical Ventilation or by In-Hospital Mortality.

		Ventilation Status			Mortality		
**Characteristic**	**All patients**	**Not ventilated**	**Ventilated**	**P-Value**	**Discharged**	**Deceased**	**P-Value**
Total patients	578	284 (53.9)	243 (46.1)	NA	467 (80.8)	111 (19.2)	NA
Median (IQR) Age (years)	62 (51-73)	59 (50-70)	62 (49-72)	0.341	58 (48-69)	76 (67-83)	<0.001
Male Sex (%)	346 (59.9)	160 (56.3)	158 (65.0)	0.043	268 (57.4)	78 (70.3)	0.014
**Admission measures, Mean (SEM)**							
Weight (kg)	84.0 (0.8)	82.9 (1.1)	85.9 (1.4)	0.089	84.2 (0.9)	83.3 (2.1)	0.691
BMI (kg/m^2)	30.3 (0.3)	29.9 (0.4)	30.8 (0.5)	0.128	30.4 (0.3)	29.9 (0.7)	0.519
Temperature > 38°C, No. (%)	72 (12.5)	30 (10.6)	41 (16.9)	0.068	61 (13.1)	11 (9.9)	0.345
HR (beats/min)	87.5 (0.7)	85.4 (1.0)	90.0 (1.3)	0.004	86.9 (0.8)	89.1 (2.0)	0.106
Systolic BP, mmHg	128.4 (0.9)	131.2 (1.3)	126.3 (1.5)	0.015	129.1 (1.1)	125.7 (2.1)	0.156
Diastolic BP, mmHg	69.6 (0.5)	70.9 (0.7)	68.9 (0.8)	0.042	70.0 (0.5)	67. 6 (1.1)	0.043
Respiratory Rate (breaths per minute)	23.7 (0.3)	21.7 (0.3)	26.3 (0.5)	<0.001	23.3 (0.3)	25.3 (0.7)	0.006
SpO_2	95.5 (0.2)	96.1 (0.2)	94.7 (0.3)	<0.001	95.5 (0.2)	95.3 (0.4)	0.563
SpO_2:FiO_2 Ratio	332.9 (5.1)	394.7 (4.83	256.3 (8.0)	<0.001	351.0 (5.3)	257.5 (11.9)	<0.001
**Comorbidities, No. (%)**							
Hypertension	304 (52.6)	143 (50.4)	125 (51.4)	0.803	234 (50.1)	70 (63.1)	0.015
Diabetes	254 (43.9)	111 (39.1)	119 (49.0)	0.023	189 (40.5)	65 (58.6)	<0.001
Coronary artery disease	100 (17.3)	42 (14.8)	34 (14.0)	0.795	65 (13.9)	35 (31.5)	<0.001
Chronic kidney disease	91 (15.7)	45 (15.8)	30 (12.3)	0.253	61 (13.1)	30 (27.0)	<0.001
COPD	76 (13.1)	33 (11.6)	25 (10.3)	0.626	50 (10.7)	26 (23.4)	<0.001
Cancer	85 (14.7)	39 (13.7)	30 (12.3)	0.605	57 (12.2)	28 (25.2)	<0.001

**Clinical or X-ray Finding, No. (%)**							
Dyspnea	422 (73.0)	184 (64.8)	204 (84.0)	<0.001	341 (73.0)	81 (73.0)	0.774
X-ray abnormality	469 (81.1)	205 (72.2)	224 (92.2)	<0.001	369 (79.0)	100 (90.1)	0.021
**Home medication, No. (%)**							
Statin	297 (51.4)	147 (51.8)	114 (46.9)	0.269	228 (48.8)	69 (62.2)	0.01
Aspirin	174 (30.1)	90 (31.7)	61 (25.1)	0.103	128 (27.4)	46 (41.4)	0.004
Anticoagulant	27 (4.7)	9 (3.2)	10 (4.1)	0.56	14 (3.0)	13 (11.7)	<0.001
**Laboratory measures, Mean (SEM)**							
Albumin (g/dL)	3.61 (0.02)	3.71 (0.03)	3.51 (0.03)	<0.001	3.65 (0.02)	3.45 (0.05)	<0.001
ALT (U/L)	64.07 (13.05)	40.34 (2.32)	96.24 (30.66)	0.002	55.97 (6.60)	97.88 (61.80)	0.276
BUN (mg/dL)	21.38 (0.77)	19.76 (0.96)	22.07 (1.35)	0.158	18.76 (0.73)	32.35 (2.25)	<0.001
CRP (mg/L)	114.2 (3.80)	83.73 (4.49)	153.3 (6.06)	<0.001	109.0 (4.23)	135.6 (8.35)	0.006
Creatinine (mg/dL)	1.46 (0.07)	1.41 (0.10)	1.52 (0.11)	0.464	1.32 (0.07)	2.02 (0.20)	<0.001
D-dimer (ng/L)	1866 (88.95)	1597 (107.5)	2129 (158.3)	0.006	1702 (91.16)	2556 (249.2)	<0.001
eGFR (mL/min/1.73m^2)	70.01 (1.26)	72.09 (1.74)	70.40 (2.02)	0.525	74.69 (1.33)	50.42 (2.67)	<0.001
Hemoglobin (g/dl)	13.18 (0.09)	13.04 (0.12)	13.50 (0.13)	0.011	13.24 (0.09)	12.91 (0.21)	0.128
LDH (U/L)	425.7 (30.12)	321.8 (7.86)	558.5 (68.05)	<0.001	392.6 (17.13)	560.4 (135.7)	0.15
MCV (fL/cell)	87.36 (0.26)	86.87 (0.39)	87.39 (0.36)	0.333	86.95 (0.28)	89.06 (0.64)	0.002
Neut:Lymph Ratio	9.17 (0.59)	6.58 (0.46)	11.92 (1.18)	<0.001	7.62 (0.46)	15.68 (2.30)	<0.001
Platelets ($\times 10^9$/L)	210.3 (3.89)	210.4 (5.58)	212.8 (6.05)	0.769	216.1 (4.31)	186.2 (8.75)	0.003
Procalcitonin (ng/mL)	1.49 (0.39)	0.62 (0.29)	2.19 (0.71)	0.114	0.81 (0.29)	4.27 (1.57)	0.012
RDW (%)	13.96 (0.08)	13.99 (0.13)	13.70 (0.10)	0.087	13.79 (0.09)	14.68 (0.18)	<0.001
Troponin T (ng/L)	38.73 (3.77)	32.62 (5.71)	42.74 (5.67)	0.228	31.19 (4.06)	69.20 (9.07)	<0.001
Troponin T>10ng/mL	0.57 (0.02)	0.48 (0.03)	0.59 (0.03)	0.02	0.50 (0.02)	0.87 (0.03)	<0.001
WBC ($\times 10^9$/L)	7.71 (0.19)	6.93 (0.19)	8.50 (0.33)	<0.001	7.61 (0.21)	8.15 (0.39)	0.26

Statistical values are for univariate logistic regression analyses. COPD = chronic obstructive pulmonary disorder; OR = odds ratio; CI = confidence interval; SD = standard deviation; ALT = alanine aminotransferase; BUN = blood urea nitrogen; CRP = C-reactive protein; eGFR =

estimated glomerular filtration rate; LDH = lactate dehydrogenase; MCV = mean corpuscular volume; RDW = red cell distribution width; WBC = white blood cell.

Supplemental Table 4. Baseline Characteristics of the Derivation and Validation Cohorts.

Characteristic	Derivation Cohort	Validation Cohort	P-Value
Total patients	578	464	NA
Median (IQR) Age (years)	62 (51-73)	66 (54-77)	0.001
Male Sex (%)	346 (59.9)	246 (53.0)	0.028
**Admission measures, Mean (SEM)**			
Weight (kg)	84.0 (0.8)	83.9 (1.0)	0.756
BMI (kg/m^2)	30.3 (0.3)	29.5 (0.3)	0.111
Temperature > 38°C, No. (%)	72 (12.5)	87 (18.8)	0.007
HR (beats/min)	87.5 (0.7)	87.0 (0.9)	0.759
Systolic BP, mmHg	128.4 (0.9)	129.0 (1.1)	0.995
Diastolic BP, mmHg	69.6 (0.5)	70.8 (0.6)	0.133
Respiratory Rate (breaths per minute)	23.7 (0.3)	22.6 (0.3)	<0.001
SpO_2	95.5 (0.2)	95.6 (0.2)	0.681
SpO_2:FiO_2 Ratio	332.9 (5.1)	349.4 (5.6)	0.028
**Race (%[*])**			
White	248 (42.9)	190 (40.9)	0.530
Black	63 (10.9)	124 (26.7)	<0.001
Hispanic	67 (11.6)	46 (9.9)	0.420
Asian	20 (3.5)	17 (3.3)	0.870
Other/Mix	121 (20.9)	56 (12.1)	<0.001
Not recorded	59 (10.2)	31 (6.7)	
**Comorbidities**			
Hypertension	304 (52.6)	284 (61.2)	0.006
Diabetes	254 (43.9)	189 (40.7)	0.314
Coronary artery disease	100 (17.3)	82 (17.7)	0.870
Chronic kidney disease	91 (15.7)	83 (17.9)	0.359
COPD	76 (13.1)	47 (10.1)	0.148
Cancer	85 (14.7)	81 (17.5)	0.234
**Clinical or X-ray Finding**			
Dyspnea	422 (73.0)	317 (68.3)	0.062
X-ray abnormality	469 (81.1)	349 (75.2)	0.148

**Home medication**			
Statin	297 (51.4)	214 (46.1)	0.092
Aspirin	174 (30.1)	144 (31.0)	0.787
**Disease severity**			
Ventilated	243 (42.0)	161 (34.7)	0.018
Deceased	111 (19.2)	99 (21.3)	0.394
**Laboratory measures**			
Albumin (g/dL)	3.61 (0.02)	3.62 (0.03)	0.774
ALT (U/L)	64.07 (13.05)	37.75 (2.41)	<0.001
BUN (mg/dL)	21.38 (0.77)	23.20 (0.89)	0.024
CRP (mg/L)	114.2 (3.80)	103.6 (4.28)	0.022
Creatinine (mg/dL)	1.46 (0.07)	1.51 (0.10)	0.224
D-dimer (ng/L)	1866 (88.95)	1959 (226.8)	0.791
eGFR (mL/min/1.73m^2)	70.01 (1.26)	65.26 (1.36)	0.005
Hemoglobin (g/dl)	13.18 (0.09)	12.46 (0.10)	<0.001
LDH (U/L)	425.7 (30.12)	383.0 (11.13)	0.277
MCV (fL/cell)	87.36 (0.26)	88.49 (0.31)	0.019
Neut:Lymph Ratio	9.17 (0.59)	8.61 (0.56)	0.233
Platelets (×10^9/L)	210.3 (3.89)	219.7 (4.56)	0.047
Procalcitonin (ng/mL)	1.49 (0.39)	1.31 (0.33)	0.683
RDW (%)	13.96 (0.08)	14.26 (0.09)	0.002
Troponin T (ng/L)	38.73 (3.77)	54.47 (7.34)	0.016
WBC (×10^9/L)	7.71 (0.19)	8.28 (0.43)	0.755

Continuous variables are presented as mean (SEM), unless otherwise noted, and categorical variables are presented as n (%). Group comparisons were performed with the Mann Whitney test, as appropriate, for continuous variables and the Fisher's exact test for categorical variables.
